# Revealing the Role of Divergent Thinking and Fluid Intelligence in Children’s Semantic Memory Organization

**DOI:** 10.3390/jintelligence8040043

**Published:** 2020-12-14

**Authors:** Clara Rastelli, Antonino Greco, Chiara Finocchiaro

**Affiliations:** Department of Psychology and Cognitive Science, University of Trento, 38068 Rovereto, Italy; antonino.greco@unitn.it (A.G.); chiara.finocchiaro@unitn.it (C.F.)

**Keywords:** semantic memory, divergent thinking, fluid intelligence, bottom-up, top-down, small-world networks, children

## Abstract

The current theories suggest the fundamental role of semantic memory in creativity, mediating bottom-up (divergent thinking) and top-down (fluid intelligence) cognitive processes. However, the relationship between creativity, intelligence, and the organization of the semantic memory remains poorly-characterized in children. We investigated the ways in which individual differences in children’s semantic memory structures are influenced by their divergent thinking and fluid intelligence abilities. The participants (mean age 10) were grouped by their levels (high/low) of divergent thinking and fluid intelligence. We applied a recently-developed Network Science approach in order to examine group-based semantic memory graphs. Networks were constructed from a semantic fluency task. The results revealed that divergent thinking abilities are related to a more flexible structure of the semantic network, while fluid intelligence corresponds to a more structured semantic network, in line with the previous findings from the adult sample. Our findings confirm the crucial role of semantic memory organization in creative performance, and demonstrate that this phenomenon can be traced back to childhood. Finally, we also corroborate the network science methodology as a valid approach to the study of creative cognition in the developmental population.

## 1. Introduction

Creativity is a central component of cognition which allows improvement in all knowledge domains, thus ensuring the flourishing of our civilization. For decades, scholars have focused on the role of creativity in developmental age and education (e.g., Guilford 1967; Lewis 2009), documenting it as a predictor of successful achievement in academic performance and future workplace performance (Torrance 1981, 1972; Gajda et al. 2017). Creative thinking has also been shown to play a key role in everyday problem solving (Wallas 1926; Runco 1994). Ergo, creative thinking abilities have been recognized as one of the major competences for the 21st century, within education and beyond (Donovan et al. 2014; Ritter and Mostert 2017). These elements make the study of children’s creativity profoundly meaningful to developmental and educational scientists (Plucker et al. 2004). Considering the relevance of this ability to human progress, we still lack a critical understanding of what makes certain people more creative than others, and this is especially true for children.

The current theories agree in seeing semantic memory—the human memory system that stores semantic concepts—as a crucial element for creative thinking, and as being involved in both bottom-up and top-down cognitive processes (Abraham and Bubic 2015; Beaty et al. 2014; Siew et al. 2019; Kenett and Faust 2019). Furthermore, the memory structure of highly creative and intelligent individuals seems to account for efficient information processing, balancing between a rigid and chaotic semantic structure, which in turn may lead to efficient and adaptive solutions to a given problem (Faust and Kenett 2014). Notably, research has largely focused on adult samples without offering clarification on the ways in which individual differences in creativity affect the structure of semantic memory among children. Therefore, in the present study, we aimed to investigate the relationship between divergent thinking (a bottom-up process), fluid intelligence (a top-down process) and semantic memory organization in children. 

### 1.1. Creativity Synergistically Relies on Bottom-Up and Top-Down Cognitive Processes

Creative cognition has traditionally been considered to be an associative process (Mednick 1962), enabling the production of novel (i.e., original, unusual, unexpected) and also useful (i.e., valuable, adaptive, appropriate) ideas (Stein 1953; Runco and Jaeger 2012; Diedrich et al. 2015). The dual-process model of creativity (Benedek and Jauk 2018) conceptualizes creative cognition as being constituted by two distinct types of thinking. In this model, as the hallmark of creativity, divergent thinking (henceforth, DT) is defined as the capacity to generate many (fluency), highly variable (flexibility), original solutions to a given problem (Acar and Runco 2014). DT has been attributed to spontaneous thought, defocused attention, and the facilitation of free-association through decreased latent inhibition (Carson et al. 2003). In experimental settings, DT is usually measured as a means of the capacity to perform open-ended tasks where there are multiple solutions to a given problem (Guilford 1950). Nevertheless, as an essential component of general intelligence, fluid intelligence (henceforth, Gf) is defined as a deductive process involving the systematic application of rules in order to draw logical conclusions (Pyryt 1998; Eysenck and Thurstone 1973; Vernon 1983; Lee and Therriault 2013). It relies on executing goal-directed behavior, and it is commonly assessed by close-ended problems that have a single correct solution (Gottfredson 1997; Guilford 1959). Gf has also been found to be associated with fluency (Batey et al. 2010), original performances on DT tasks (Benedek et al. 2012; Silvia and Beaty 2012; Forthmann et al. 2019; Jung and Haier 2013; Karwowski et al. 2016), and the adoption of creative strategies (Nusbaum and Silvia 2011).

Several studies related DT to cognitive flexibility both in adults (Pan and Yu 2018; Benedek et al. 2012) and children (Krumm et al. 2018). Indeed, the flexibility of thought facilitates the production of innovative ideas by switching from one concept (or category) to another and forming remote connections (Pan and Yu 2018). The development of DT in children has been a well-studied topic in the field of education for more than fifty years (Smith and Carlsson 1985; Kim 2011). However, the empirical findings regarding children’s creativity have not been consistent in the literature (Gralewski et al. 2017; Said-Metwaly et al. 2020). For pre-schooled children (2 years old) (Bijvoet-van den Berg and Hoicka 2014), the individual differences in DT performances seem to increase across grade levels (e.g., Sak and Maker 2006), and tend to continue until about age 40, followed by a “systematic maturational declines” (McCrae et al. 1987). Other studies, however, have reported that DT follows an irregular developmental trajectory with significant drops (e.g., Charles and Runco 2001; Kim 2011), especially between the ages of 8 and 10, when logical thinking becomes fully functional (Torrance 1968; Charles and Runco 2001). In regard to Gf, a study found positive associations between creativity and Gf in children aged from 8 to 14 years, as measured by the Verbal TTCT and both the Kaufman Brief Intelligence Test and Raven’s Standard Progressive Matrices (SPM) (Krumm et al. 2014). Another recent work investigated the role of executive function on creativity, demonstrating that—after controlling for the children’s level of intelligence—some specific executive functions, such as shifting and inhibition, make a significant contribution to creativity (Krumm et al. 2018). Consequently, the current theories mainly agreed in seeing creative cognition as either a bottom-up or top-down cognitive process (Beaty et al. 2014). On the one hand, the bottom-up process suggests that differences in creative cognition can be mediated by changes in the structure and access to concepts within the semantic memory (Kenett et al. 2014; Kenett et al. 2016a; Rossmann and Fink 2010; Schilling 2005; Mednick 1962; Kenett and Faust 2019). The bottom-up account of creative cognition relies on a distinguished model in the literature coined by Mednick, the so-called “associative theory of creativity” (Mednick 1962). In this framework, the author advocates that the concepts’ hierarchy in the semantic memory of creative individuals is more “flat” (more associations and weak links) rather than “steep” (fewer and stronger associative links), which enable to perform faster remote associations as compared to less creative individuals (Kenett 2018; Rossmann and Fink 2010; Kenett et al. 2014; Mednick 1962). The top-down process, on the other hand, taps individual differences in creative cognition within the ability to exert control over attention (Beaty et al. 2017, 2014; Nusbaum and Silvia 2011), and it relates to convergent thinking, executive functions, and particularly fluid intelligence (Benedek et al. 2014; Jauk et al. 2013; Frith et al. 2019; Silvia 2015; Prabhakaran et al. 2014; Silvia and Beaty 2012; Nusbaum and Silvia 2011; Lee and Therriault 2013). Supported by the executive theory of creative thought, the top-down process is thought of as being preponderant during the selection and evaluation of an idea, allowing a more efficient memory retrieval and implementation (Beaty and Silvia 2012; Dietrich 2004; Benedek et al. 2014; Krumm et al. 2018), and mediating switching ability (Nusbaum and Silvia 2011), shifting (Lee and Therriault 2013), and updating (Beaty et al. 2014). Although the bottom-up and top-down processes appear to be competing, a growing body of research indicates that both processes synergistically contribute to creative ability (Benedek et al. 2014; Benedek and Jauk 2018; Frith et al. 2019). Crucially, the structure of the semantic memory represents a shared fundamental element of both processes (Abraham and Bubic 2015; Beaty et al. 2014; Siew et al. 2019; Kenett and Faust 2019). 

### 1.2. Small-World Structure of Semantic Memory

Semantic memory is a human memory system that stores semantic categories and concepts, generalizing across distinct facts from our lives, independent of time or context (McRae and Jones 2013; Patterson et al. 2007). According to the spreading activation model of Collins and Loftus (Collins and Loftus 1975), the semantic memory space is constituted by concepts (words) as nodes and edges defined by the strength of shared associations. Thus, nodes are connected according to a principle of semantic similarity, i.e., the greater the semantic proximity between the concepts and the force connecting them, the more the semantic correlation increases while their distance reduces (Collins and Loftus 1975). Currently, Network Science approaches represent a reliable and well-established tool which enables us to quantitatively analyze the role of semantic memory at a cognitive level. Network Science has been applied by an increasing amount of research in the cognitive domains (e.g., Baronchelli et al. 2013; Collins and Loftus 1975; He et al. 2020; Patterson et al. 2007; Siew et al. 2019). One of the models that is used the most in order to examine complex systems is the Small-world Network model (SWN) (Kleinfeld 2002; Watts and Strogatz 1998). Small-world networks are considered efficient because they allow fast communication between any two nodes in a network even though they have few edges (Humphries and Gurney 2008). The main features of SWN include:

1. The clustering coefficient (CC), which suggests that nodes that are near-neighbours tend to co-occur and to be related. A higher level of CC denotes a better local organization, showing more interconnection within the network.
2. The Average Length of the Shortest Path (ASPL), which indicates the quantity, on average, of the shorter steps between two pairs of nodes. ASPL also characterizes the strength of the association, which is a key factor in the spread of the activation (Anderson 2013; Siew et al. 2019; Kenett 2018); thus, a lower ASPL might improve the chances of reaching a wider number of connections.
3. The modularity index (Q) allows us to quantify the ways in which a network is divided into sub-networks; the larger the modularity the better the network breaks apart into sub-communities (Newman 2006; Fortunato 2010).
4. Finally, the ‘small-world-ness’ measure (S) can be considered to be an index of a network’s efficiency, flexibility and chaos. A small-world network is characterized by high local connectivity (higher CC) and short global distances between nodes (lower ASPL). Therefore, the S measure can be quantified as the ratio of a network’s CC over a network’s ASPL (Humphries and Gurney 2008), providing efficient diffusion searching and a more efficient information retrieval through semantic space (Marupaka and Minai 2011; Anderson 2013). Semantic memory structure has been posed in a continuum between rigidity (order) and flexibility (chaos) modelling thought processes. On the one end of this continuum is an extreme state of rigidity, as seen in the semantic network of people with high-functioning autism (Kenett et al. 2016b). By contrast, exceedingly small-world-ness properties of the network may contribute to more improper associative relations, raising the likelihood of chaos (Spitzer 1997; Faust and Kenett 2014). Therefore, small-world networks seem to account for efficient semantic processing via a balance between rigid and chaotic semantic structures (Faust and Kenett 2014).

The SWN model has already offered valuable insights into the nature of language (Siew 2018; Steyvers and Tenenbaum 2005; Cancho and Solé 2001), language acquisition and development (Beckage et al. 2011), memory retrieval (Zemla and Austerweil 2019), information organization (Borge-Holthoefer and Arenas 2010; De Deyne et al. 2019), learning (Karuza et al. 2016), and across an individual’s lifespan (Zortea et al. 2014; Wulff et al. 2019). In this context, previous studies have provided evidence towards changes in the semantic memory organization across and individual’s lifespan, pointing out that children tend to have distinct semantic memory profiles compared to those of adults (Wulff et al. 2018; Beckage et al. 2010; Dubossarsky et al. 2017; Wulff et al. 2019; Zortea et al. 2014). Broadly, these researchers suggested that, whilst the size of the network seems to be increasing throughout life up to adulthood (Beckage et al. 2010), children’s networks seem to show fewer nodes, connections and clusters, and longer ASPL (Dubossarsky et al. 2017; Wulff et al. 2019; Zortea et al. 2014).

### 1.3. Semantic Memory Structure, Divergent Thinking Creativity and Fluid Intelligence

Concerning creativity, a limited but growing number of studies are examining the role of the semantic memory structure using small-world network models (Kenett and Faust 2019; Kenett et al. 2014; Schilling 2005; Marupaka et al. 2012). A few studies have examined the ways in which variations in semantic memory structure lead to individual differences in creativity and fluid intelligence (Gray et al. 2019; Benedek et al. 2017; Kenett et al. 2016a; Kenett et al. 2014). One of the first, albeit recent, empirical works that has shed light on these relations using network science methods comes from Kenett et al. (Kenett et al. 2014). The authors used a group-based Network Science approach (Kenett et al. 2013) which provided empirical evidence for the associative theory of creativity (Mednick 1962). In their study, low and high creative ability individuals’ groups underwent a free association task on 96 words. The semantic networks of the groups were estimated and compared, computing edges between the pairs of nodes on the overlap of the associative responses generated to each of them. As a result of the analysis, the semantic memory networks of the individuals with high creative ability were more flexible, showing higher CC, shorter ASPL, lower Q, and a higher S compared to the networks of the low creative individuals (Kenett et al. 2014). The flexible properties of the creative semantic network were interpreted by the authors as enabling more efficient retrieval strategies when connecting remote associations, via clustering (high CC) and switching (low ASPL) processes. Particularly, a shorter ASPL may provide a quicker exploration within the network for remote semantic concepts. In this regard, a number of studies offered strong evidence that highly creative individuals use efficient search processes that reach further and weaker connected concepts (Kenett and Austerweil 2016; Gray et al. 2019; Rossmann and Fink 2010). Additionally, researchers gained even more evidence about the flexible properties of the creative semantic memory structure from a study that probed the robustness of the network with response to targeted attacks within a percolation theory framework (Kenett et al. 2018). The semantic network structure of highly creative people resulted in higher robustness to percolation analysis, highlighting the flexible properties of the network of high creative ability individuals as compared to low creative ability individuals (Kenett et al. 2018). 

Notably, a recent study examined the relationship of Gf, creative ability and semantic memory structure (Kenett et al. 2016a). In that study, a semantic verbal fluency task was administered to the participants, and was used to construct the semantic networks at a group level. The participants were divided into two groups based on their performance on intelligence and creativity measures. As a result of the comparison for all of the groups, the analysis revealed that, although Gf was more related to the structural properties of the semantic network (higher ASPL and Q values), creativity was more related to the flexible properties of the network (higher S value and lower Q values). Crucially, the group with higher creativity and intelligence was found to be characterized by both the flexible and structured properties of the lexical network. The reported results provided support for previous research that revealed that individuals with low latent inhibition and high IQ demonstrate a remarkable creative performance (Carson et al. 2003). Even more so, their results contributed to the validation of the ‘controlled chaos’ theory of creativity (Bilder and Knudsen 2014) by probing its flexible, chaotic property (accounting for originality), and its constrained, structured property (accounting for appropriateness) (Kenett et al. 2014; Siew et al. 2019; Faust and Kenett 2014; Kenett et al. 2016b). Finally, the above-mentioned results were partially replicated by Benedek and colleagues (Benedek et al. 2017), who implemented a novel method for the construction of individual semantic networks based on relatedness ratings. The authors found that the DT creativity networks exhibited stronger clustering with shorter average distances between concepts (indicating higher small-world-ness) and lower modularity. On the other hand, no significant association of semantic memory and Gf was found in this experiment, possibly because—as suggested by the authors—an expedited version of the Gf task was used. To summarize, these studies, although preliminary, acknowledge a variation in semantic memory structure due to the contribution of individual differences in creativity and intelligence, with creativity being more involved in a flexible and chaotic organization (higher S and lower Q) and intelligence being associated with a more rigid structural property of the semantic network (higher Q and ASPL).

### 1.4. The Present Study

The above-mentioned studies point out evidence that both DT (bottom-up) and Gf (top-down cognitive) are fundamental components of creative thinking, in children as well as in adults. However, while the foregoing research offered profound insights regarding the relationship between bottom-up/top-down cognitive processes and the semantic memory structure, these studies have mainly focused on the adult population and, to our knowledge, there are no studies that have been carried out with children. Moreover, given the differences between the semantic memory of adults and children, and the developmental nature of the children’s creativity and cognitive functions (Arán Filippetti and Krumm 2020; Diamond 2013), the investigation of these constructs in childhood becomes important, as their configurations could be different from those proposed for adults. Thus, the present study aimed to examine the ways in which DT (bottom-up process) and Gf (top-down process) are related to the structure of semantic networks in children. Here, DT was assessed using the well-known Alternative Uses method (Torrance and Presbury 1984), and G*f* was evaluated using the Standard Progressive Matrices (SPM) (John and Raven 2003). We divided the sample in half to form groups of high and low DT and Gf groups, respectively. We constructed the semantic memory networks based on participants’ responses in a verbal fluency task, and we examined it using Network Science methodology at the group level (Kenett et al. 2013). 

Hence, if the organization of children’s semantic memory is comparable to that of adults, we hypothesized that the semantic network of the highly creative children group tends to have a structure that is more interconnected but less structured (shorter ASPL and higher CC), with higher flexible and chaotic properties (higher S and lower Q) compared to the less creative group. These predictions would be in line with the previous findings that support the bottom-up account of creativity (Kenett et al. 2014; Kenett et al. 2016a; Mednick 1962; Rossmann and Fink 2010; Schilling 2005; Kenett and Faust 2019). According to the top-down account (Unsworth et al. 2011; Benedek et al. 2017; Kenett et al. 2016a; Faust and Kenett 2014), however, we expected to find a more structured and rigid (longer ASPL and higher Q) semantic network in the higher intelligence group than in the lower intelligence group. 

## 2. Materials and Methods 

### 2.1. Participants

The participants were recruited from a primary school in Rovereto (Italy). Out of 61 healthy volunteers, 58 native language speakers were included in the analyses, the mean age was 10.01 years (SD = 0.31), and 55.2% were male. The experiment was conducted in the classroom (completed in groups of ~20 participants) under the supervision of the authors; the participants were spaced apart. The behavioural measures were administered as a paper and pencil task. All of the participants and their parents gave written informed consent. The study protocol was approved by the Human Research Ethics Committee of the University of Trento.

### 2.2. Behavioural Measures

#### 2.2.1. Vocabulary Knowledge

The subscale ‘Verbal meaning—words’ from the Primary Mental Abilities (PMA) test (Eysenck and Thurstone 1973) was chosen as a measure of vocabulary knowledge. The children were presented with a list of 13 words, and were asked to choose—among four alternatives—the word with the same meaning. The correct responses were summed up to give a final score of the vocabulary knowledge. 

#### 2.2.2. Written Words per Minute (WPM)

Words per minute were used as a measurement of the children’s writing speed. Studies compiled by (Amundson 1995) showed that copying rates using handwriting at the end of elementary school, at the 5th and 6th-grade level, are about 10 to 12 WPM. Here, all of the participants copied a standard sentence in their best handwriting repeatedly for one minute. The sentence is taken from the speed-copy subtest of the DGM-P (DGM-P: graph-motor and postural difficulties of handwriting test) (Borean et al. 2012): “*L’elefante vide benissimo quel topo che rubava qualche pezzo di formaggio*” [The elephant saw very well that mouse stealing some pieces of cheese]. Illegible words, the final word (if it was incomplete), and punctuation marks were excluded from the score.

#### 2.2.3. Semantic Verbal Fluency (SVF)

A widely used neuropsychological measure that identifies the ability of lexical control and the structure of semantic memory is semantic verbal fluency (Ardila et al. 2006). This test requires the generation of words according to a specific semantic category. Among the different semantic categories previously used in the literature to test SVF, that of “animals” appears to be the most frequently-used, as it is linked to its frequent early and extensive knowledge, with a well-defined taxonomy showing minor differences across different languages (Goñi et al. 2011). According to the standard procedure, the participants had one minute to generate as many animal category members as they could think of. For each participant, repetitions and non-category members were excluded from further analyses. 

#### 2.2.4. Divergent Thinking (DT)

Divergent thinking tests measure the ability to generate multiple different solutions (Carroll and Guilford 1968), and are widely used to test creativity (Cropley 2000; Torrance and Presbury 1984). The most-used task in the literature is the Alternative Uses Task (AUT) (Torrance and Presbury 1984). In it, the participants were asked to list as many different and creative uses for the object “brick” as they could think of. Creative performance during the AUT is reflected in an Overall Divergent Thinking (ODT) index, which is calculated by scoring the participants’ performance on three major scales:

Cognitive Flexibility: this measures the number of different categories of ideas, and corresponds to the number of different concept categories that a person uses (Amabile 1983). Here, in designing the list of concept categories for the word “brick”, two trained raters collaborated. Thus, each rater allocated each idea produced by the participants to one concept category from a predefined list. Subsequently, one of the raters assigned each idea to a category on the predefined list of concept categories. Finally, the overall number of distinct categories used by each participant was determined. 

Fluency: this is a measure of creative production representing the total number of ideas generated. In order to assign a fluency score, the total number of complete and non-redundant ideas from a participant are included. 

Creativity: recently, and in part motivated by Amabile’s Consensual Assessment Technique (CAT) (Amabile 1983), researchers have advocated the use of subjective scoring approaches, according to which a panel of experts rates the generated uses directly on creativity (Scott et al. 2004). Here, two trained raters performed the creativity score for each idea, ranging from not at all creative (=1) to very much creative (=5). In scoring creativity, we considered the two essential criteria for the generation of creative ideas: novelty and usefulness. Each idea was scored as the average given by the raters. The inter-rater reliability of the ratings was calculated using intraclass correlation coefficient (ICC) analysis for consistency. There was a substantial agreement between the two raters using the two-way random effect models (ICC = 0.71). A creativity sum score was calculated per participant by adding up the scores of the ideas a participant generated. According to previous studies (e.g., de Dreu et al. 2012; Jung et al. 2015), the use of a sum score is based on the assumption that creativity increases with the number of ideas produced (Osborn 1963; Beaty and Silvia 2012). Since the creativity index can be correlated with fluency, as the number of ideas might be a confounder of the ideas’ quality, mean scores were calculated for each participant by dividing the creativity index score by the fluency score. 

Along the same line, the overall divergent thinking score (ODT) was calculated by adding up the creativity sum score divided by fluency and cognitive flexibility (see Ritter and Ferguson 2017).

#### 2.2.5. Fluid Intelligence (Gf)

The Standard Progressive Matrices (SPM) (John and Raven 2003) are one of the most frequently used and well-validated tests for the measurement of fluid intelligence (Gf); they are composed of 60 items divided into 5 series of 12 items each. Each item requires the completion of a series of figures with the missing one, compared to a presented model, and according to a criterion of increasing difficulty. The model figures include graphic patterns that change from left to right and from top to bottom; the participant must understand the underlying logic and apply it in order to reach the solution. 

### 2.3. Data Analysis

#### 2.3.1. Group Construction

Since an unbalanced sample size between the experimental groups could produce uncorrected results, as in previous studies (Kenett et al. 2016a) the participants were divided based on the median of either the DT or Gf score. This method allows us to include the same number of participants in the two groups (low vs. high) for each independent variable (DT and Gf). In the output, we had two different groups for DT and for Gf, respectively: low DT; high DT; low Gf; high Gf.

#### 2.3.2. Unique Word-Associations

The numerical difference of unique responses generated by each group was examined using McNemar’s chi-squared test (Agresti 2003). The total number of unique responses within each group were summed-up; its greater number might suggest a greater depth of knowledge for the semantic category of animals (Christensen et al. 2018).

#### 2.3.3. Semantic Network Construction

The semantic fluency data were analyzed through the implementation of a network science approach recently developed by Kenett and colleagues (Kenett et al. 2013) which has been applied extensively (see Borodkin et al. 2016; Christensen et al. 2018; Kenett et al. 2016a; Kenett et al. 2014). In this framework, the data are modelled as a network in which the nodes represent the concept (e.g., dog) and edges represent associations between two concepts. This association reflects the trend of the participants to generate a word “b” (e.g., cat) given that a word “a” is generated (e.g., dog). Notably, the following approach cannot examine networks as individual differences among participants; instead, it is able to examine the group-based general difference of the network structure, based on how often the responses co-occur across the groups (Kenett et al. 2013). The whole semantic network analysis pipeline is described below. First, during the preprocess, we excluded idiosyncratic responses and non-words. Next, we controlled for other possible confounders (i.e., converted plural words into singular), and finally we translated the word responses in English from Italian. In order to analyze the dataset, we first standardized the data into a matrix *j* × *i,* with each column representing the unique word responses (e.g., dog) given by the entire sample, and with each row containing all the responses of a single participant. In this way, the participants’ responses were encoded as 1 when participant *j* provided the word *i,* and 0 when that participant did not. Next, we created four different matrices according to the respective groups of low/high DT and low/high G*f*. All of the unique animal responses were equated between the low and high groups of DT and G*f* separately. Here, only the responses generated by two or more participants in both groups were included (Kenett et al. 2013, 2016). This step allows us to compare the networks since they are constructed with the same nodes, thus controlling for confounding factors (e.g., differences in nodes or edges) (Christensen et al. 2018; Wijk et al. 2010). Thus, we constructed a word-correlation matrix between all the pairs of words for each group. Here, we applied the cosine similarity measure with the following formula (using the SemNeT package; (Christensen and Kenett 2019) https://github.com/AlexChristensen/SemNeT):

(1) $$cos=\frac{\Sigma_{j}^{n}=1^{A_{j}B_{j}}}{\sqrt{\sum_{j=}^{n}1^{A_{j}^{2}}}\sqrt{\sum_{j=}^{n}1^{B_{j}^{2}}}},$$

where *A_j_* indicates the column of response *a,* and *B_j_* indicates the vector of response *b*. Despite the fact that Pearson’s correlation was used in prior work (Kenett et al. 2014; Kenett et al. 2016a), we followed the reasoning of Christensen and colleagues (Christensen et al. 2018) according to which the resulting associations using the cosine similarity are all positively valued (ranging from 0 to 1), giving the advantage of not assuming a negative association between two responses. The obtained word similarity matrix is an n × n adjacency matrix of a weighted, undirected network, where n depicts the nodes (word responses) and the cells depict the similarity between all of the pairs of words. In order to overcome the loss of information given by spurious associations, we used the Triangulated Maximally Filtered Graph (TMFG—Massara et al. 2016) method which, in the construction of a sub-network, is able to remove spurious connections and retain high correlations within the original graph (see Kenett et al. 2011). The TMFG filtering method was applied using the ‘NetworkToolbox’ package (Christensen 2019; Kenett et al. 2014) in R (Rstudio Team 2020). Finally, we further binarized each group-similarity network in order to obtain in output an unweighted, undirected network.

#### 2.3.4. Network Measures Estimation and Validity

The following network parameters were calculated for each network using the SemNeT (Christensen and Kenett 2019) and NetworkToolbox (Christensen 2019) packages in R: the clustering coefficient (CC) (Watts and Strogatz 1998) the average shortest path length (ASPL), the modularity index (Q) (Newman 2006), and the small-world-ness measure (S) (Humphries and Gurney 2008). Based on previous studies (Borodkin et al. 2016; Christensen et al. 2018; Kenett et al. 2014), we empirically examined the validity of our findings by applying two reciprocal approaches. Firstly, in order to statistically test whether the network parameters did not result from the null hypothesis of a random network, several Erdös–Rényi random networks were simulated (N = 1000) with the same number of nodes and edges for each network group (Erdos and Rényi 2011). For these random networks, we calculated all of the network measures (CC, ASPL, Q and S). Thus, we used a one-sample Z-test to compare each random reference distribution with the network measures for each group. Secondly, in order to compare low and high DT and low and high G*f* networks, we simulated many partial random networks by applying the bootstrap method (Efron 1979). Unlike others, the bootstrap method does not rely on any statistical assumptions, but rather on computational ability to simulate data (Shalizi 2010), giving to this method a more significant strength. The bootstrap procedure (without replacement Bertail 1997; Shao 2003) consists of the random selection of half of the responses (nodes) of each semantic network to construct partial sub-graphs and compute the network measures for each sub-graph. In this way, two networks were considered different from each other if any sub-network, consisting of the same nodes in both networks (low vs. high), is also different. Thus, this approach enables the statistical analysis of the difference between any network pair. Furthermore, we analyzed the reliability of this method through the construction of graded partial semantic networks for each group by selecting 60%, 70%, 80%, and 90% of the nodes (Epskamp et al. 2018). We simulated 1000 realizations for each graded partial network and each group, calculating different measures, for the DT networks (CC, ASPL, Q, and S) and for the G*f* (ASPL, Q). Here, we applied an independent *t*-test of these measures for each graded partial bootstrapped network, comparing the low vs. the high DT networks and the low vs. the high G*f* networks separately, using the respective measures of interest. 

## 3. Results

### 3.1. Correlation Analysis of the Overall Divergent Thinking (ODT) 

In order to examine the relations between the divergent thinking measures, we conducted a Spearman correlation analysis between the ODT and the other indices’ scores. Since the AUT variables Fluency, CreativitySum, and Flexibility were skewed, we computed the log-transformation on these scores. The results revealed a comprehensive positive significant correlation with all being *p* < 0.001, as shown in Table 1. This finding positively relates the ODT index to cognitive flexibility, fluency, and the raters’ score of creativity. 

### 3.2. Groups Construction Based on Fluid Intelligence (Gf) and Divergent Thinking (DT) Scores

We divided the starting pool of 58 participants based on the median of either their ODT (DT) or SPM (Gf) score and obtained two groups of 29 individuals each, depending on the selected variable. The two groups of low vs. high DT and low vs. high Gf did not differ significantly in age, gender, vocabulary knowledge, or written words per minute. Nevertheless, they differed significantly as to the respective independent variable (Gf/DT), in that the high DT group had significantly higher scores on the ODT measure compared to the low DT group (t = 11.464, *p* = < 0.001, d = 3.01), while the high Gf group had a significantly higher score on the fluid intelligence measure (SPM) compared to the low Gf group (t = 10.224, *p* = < 0.001, d = 2.68). The descriptive statistics and *t*-test results are presented in Table 2.

### 3.3. Semantic Memory Networks

All of the unique animal responses were equated between the low and high groups of DT and Gf, respectively. The DT groups resulted in 31 word-association responses, while the G*f* groups obtained 33 word-association responses. We constructed the semantic networks by applying the TMFG filtering method to the word similarity matrix. Next, the different networks’ properties of the animal category lexical networks were calculated from the filtered similarity matrix, and were compared. The networks were visualized using the force-directed layout of the CYTOSCAPE software (Shannon et al. 2003) (version: 3.8.1). The 2D graphs represent nodes (word category members) as circles and links between them, understood as the symmetrical edge (i.e., bidirectional) similarities between two nodes. Firstly, the simulated random networks analysis showed that all of the empirical network measures for each (DT/G*f*) group significantly differed from their simulated random measures (all *p*’s < 0.001). Moreover, in order to examine the statistical differences between the low and high Gf or DT groups, unpaired *t*-tests were computed for each partial bootstrap analysis. The results from the network analysis revealed qualitative and quantitative differences between the levels (low/high) of either the DT and Gf networks’ structures.

#### 3.3.1. Divergent Thinking (DT) 

The semantic network of the high DT group showed lower structural (ASPL = 2.13 and Q = 0.43) and higher flexibility (S = 2.95) values compared to the low DT group (ASPL = 2.36; Q = 0.45; S = 2.79), as shown in Table 3. 

**Figure 1:**
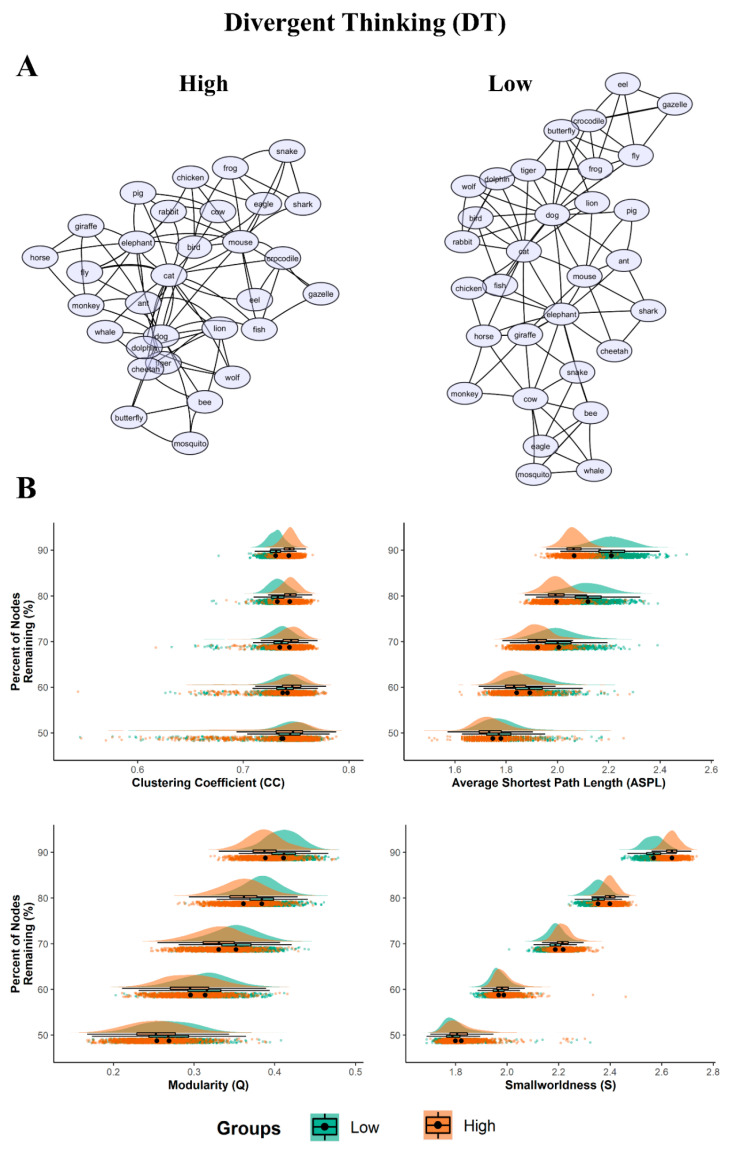
Semantic networks and bootstrapped results for DT. (**A**) 2D graph visualizations of the DT groups (high and low). The graphs are unweighted and undirected, with nodes (word responses) represented as circles, and the links between them (edges) represented as symmetrical similarities between two nodes; (**B**) Plots of the bootstrapped partial network measures (1000 samples per nodes remaining percentage). The density plots are above the scatterplots (individual dots depict a single sample), with a black dot representing the mean. The y-axis denotes the percentage of nodes remaining, with the legend of the DT groups (low and high) below the plots. All *p* < 0.001.

Indeed, the low DT network appeared to be more spread out and more structured (divided into sub-groups) than the high DT networks. Conversely, the high DT lexical network was more compact, with a reduced distance between associations (Figure 1 section A). 

The simulated random networks analysis showed that all of the empirical network measures for each DT group significantly differed from their simulated random measures (all *p*’s < 0.001, see Table 3). The bootstrap analyses revealed the prevailing statistically significant differences in structure between high/low DT networks (Figure 1, section B and Table 4). 

Precisely, the ASPL was significantly smaller for the partial networks of the high DT compared to the low DT group. The effect size ranged from moderate (d = 0.41; when 50% of the nodes were dropped) to very large (d = 2.35). In the same way, the Q was significantly smaller for the partial networks of the high DT compared to the low DT group, with the effect size ranging from moderate to large (d = 0.42 to 1.02). The partial networks of the high DT group had a significantly higher CC across the bootstrapped samples compared with the partial networks of the low DT. However, while when 60% to 90% of the nodes were dropped the effect sizes across the bootstrapped samples varied from small to very large (d = 0.23 to 1.67), when 50% of the nodes were dropped the CC did not reach statistical significance (with t = 0.926, *p* = 0.354, d = 0.041). Finally, the partial networks of the high DT group showed a significantly higher S across the bootstrapped samples compared to the partial networks of the low DT. The effect size varies from moderate to very large (d = 0.36 to 2.10). The results thus showed robust differences in semantic network organization between the low and high DT groups. 

#### 3.3.2. Fluid Intelligence (Gf)

The semantic network of the high Gf group showed longer ASPL (2.21) and higher Q (0.41) compared to the low Gf group (ASPL = 2.10; Q = 0.40), as shown in Table 5. 

**Figure 2:**
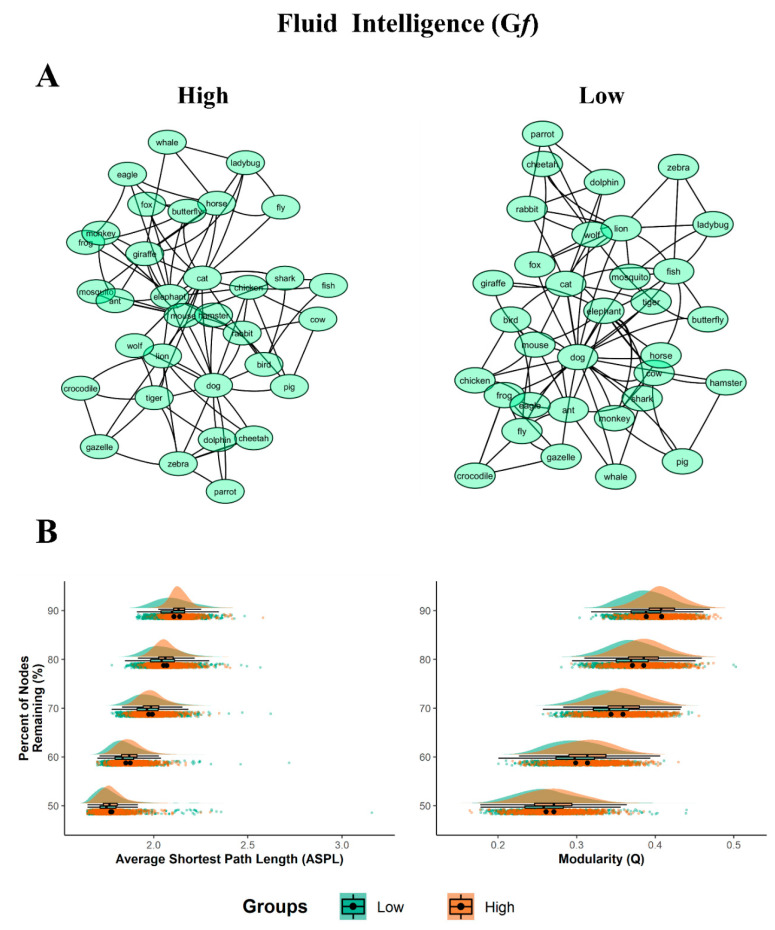
Semantic networks and bootstrapped results for Gf. (**A**) 2D graph visualizations of the Gf groups (high and low). The graphs are unweighted and undirected, with nodes (word responses) represented as circles, and the links between them (edges) represented as symmetrical similarities between two nodes. (**B**) Plots of the bootstrapped partial network measures (1000 samples per nodes remaining percentage). The density plots are above the scatterplots (individual dots depict a single sample), with a black dot representing the mean. The y-axis denotes the percentage of nodes remaining, with the legend of the Gf’ groups (low and high) below the plots. All *p* < 0.001, with the exception of ASPL at 50% which were not significant.

The high G*f* network appeared to be more structured (divided into sub-groups) than the high DT networks (Figure 2 section A). 

The simulated random network analysis showed that the majority of the empirical network measures for each G*f* group significantly differed from their simulated random measures (all *p*’s < 0.001; see Table 5). The bootstrap analyses revealed the prevalence of statistically significant differences in structure between high/low Gf networks (Figure 2, section B and Table 6). 

As to the Gf groups, the ASPL was significantly longer for the partial networks of the high Gf group compared to the low Gf group. However, while the effect sizes across the bootstrapped samples were moderate when 60% to 90% of the nodes were dropped (from d = 0.30 to d = 0.39), when 50% of the nodes were dropped the ASPL did not reach statistical significance, with t = −1.355 and *p* = 0.176, d = 0.06). Although small differences emerged, the Q measure was significantly smaller for the partial networks of the low Gf compared to the high Gf group, with the effect size ranging from small to moderate (d = 0.28 to 0.74). Taken together, the effect sizes—ranging from small to medium-large—showed considerable differences in the semantic network organization between the low and high Gf groups.

### 3.4. Unique Word Association

The semantic fluency data were preprocessed and organized into a matrix, with each column representing the unique concept across the sample (129 unique responses) and each row containing all of the responses given by a single participant. The total number of unique responses generated by the groups was 105 in the high DT (45 words were not given by the low DT group), 84 in the low DT (24 words were not given by the high DT group), 100 in the high Gf (38 words were not given by the low Gf group), and 91 in the low Gf (29 words were not given by the high Gf group). In order to test the difference in the proportion of these responses given by each group (low vs. high), we applied McNemar’s test. The percentage of unique responses that originated in the high DT group (81.4%) was significantly higher compared to the percentage in the low DT group (65.1%), with χ^2^ = 5.797, *p* = 0.016, φ = 0.212. The unique responses of the high Gf group (77.5%) were quantitatively more compared to those of the low Gf group (70.5%), although this difference did not reach statistical significance (χ^2^ = 0.955, *p* = 0.328, φ = 0.09).

## 4. Discussion

### 4.1. Children’s Semantic Networks

The present study is the first, to the best of our knowledge, to examine the relationship between semantic network structure, divergent thinking creativity and fluid intelligence among children. We started from the commonly-shared assumption that the organization of the semantic memory represents a fundamental component of creative cognition, mediating bottom-up and top-down cognitive processes (Beaty et al. 2014; Abraham and Bubic 2015; Kenett and Faust 2019). The participants were divided into two groups, twice, based on the median of both divergent thinking and fluid intelligence task’s performances. We applied a recently-developed method (Kenett et al. 2013) based on a Network Science approach in order to examine group-level semantic memory networks. The networks were constructed on the basis of a semantic fluency task. The four networks, of high vs. low DT groups and high vs. low Gf groups, were then compared. As a last step, we empirically examined the validity of our findings by applying two complementary approaches. In the former, we tested the null hypothesis of no differences in semantic networks across the groups by simulating several Erdös–Rényi random networks (Erdos and Rényi 2011) and using a one-sample Z-test to compare each random reference distribution with the network measures for each group. In the latter, we simulated many partial random networks by applying the bootstrap method. We applied an independent sample *t*-test on the computed measures for each partial network constructed from subsets of nodes (selecting 60%, 70%, 80%, and 90% of the nodes), comparing low vs. high DT/Gf networks, respectively (for similar procedures see Kenett et al. 2014; Christensen et al. 2018). These analyses revealed consistent differences in both variables. Firstly, the semantic network of the children belonging to the high creative DT group displayed a better local organization of associations (higher CC), showing more interconnection with less structured properties (lower ASPL and Q) and greater flexibility (higher S) relative to the semantic network of the low creative DT group. Secondly, the semantic network of the high Gf group exhibited a more structured organization with less-interconnected nodes (higher ASPL) and a more structured network (higher Q) compared to the low Gf group. The partial network analysis verified these results, corroborating the relationship of creative divergent thinking and fluid intelligence with the structure of the children’s semantic network. 

#### 4.1.1. Divergent Thinking

As predicted, the analysis revealed that the full network of the high DT children’s group had smaller ASPL and Q values, while the CC and S values were higher than those in the low DT group. The statistical analysis from the partial bootstrapped networks supports the findings for the full networks, consistently across the bootstrapped realizations. Here, the effect size (which ranged from small to very large) increases as the nodes are retained (from 50% to 90%), with the only exception of the CC value that was not significant when 50% of the nodes were retained. These findings reveal robust differences in the organization of the semantic memory in children. We interpreted these results as showing that high DT children display more efficient retrieval strategies, via clustering (high CC) and switching (low ASPL) processes, compared to the low DT children. More precisely, the larger CC in the network of the high DT group could provide a greater local organization, while the shorter ASPL suggests greater interconnectivity between nodes, facilitating remote associations. In this regard, several studies provided strong evidence that highly creative individuals employ faster search processes that reach further and weaker-connected concepts both in children and adults (Kenett and Austerweil 2016; Gray et al. 2019; Rossmann and Fink 2010; Kenett 2018; Prabhakaran et al. 2014; Pan and Yu 2018; Bijvoet-van den Berg and Hoicka 2014). Coherently, characterized by short ASPL and high CC, the semantic network of children in the high DT group showed greater small-world properties compared to the low DT network. Small-world properties give the network a more flexible structure, enabling a more efficient search through semantic space (Marupaka and Minai 2011), and facilitating the search and retrieval of associations in memory (Anderson 2013). Furthermore, the flexible properties of the creative semantic network were corroborated from the smaller Q exhibited by the high DT group’s network, which suggests a less rigid network.

#### 4.1.2. Fluid Intelligence

The analysis revealed that the full network of the high Gf group had a higher ASPL and Q than the low Gf group, in line with our prediction. The statistical analysis from the partial bootstrapped networks supports the outcomes for the full networks, consistently across the bootstrapped realizations. The effect size (which ranged from small to medium-large) increases as the nodes are retained (Q values increase from 50 to 90%; ASPL values increase from 60 to 90%).The larger ASPL and Q in the network of the high Gf group indicates a more structured network, which replicates the previous findings of Kenett and scolleagues (Kenett et al. 2016a). Indeed, the greater modularity of the network has already been found to be related to intelligence and language development (Borodkin et al. 2016; Kenett et al. 2016a). The relation between Gf and a more structured semantic memory has been linked to the ability of the individual to easily switch between smaller modules, thereby defeating the common solutions to a given problem (Nusbaum and Silvia 2011; Unsworth et al. 2011). In support of this argument, current theories have demonstrated that modularity represents a key aspect in the flexibility of thought by constraining the spread of activation over semantic networks (Kenett et al. 2014; Siew et al. 2019; Faust and Kenett 2014; Kenett et al. 2016b).

### 4.2. Unique Number of Responses

The present findings showed that both the high DT and high Gf group generated a higher number of unique responses compared to the low DT and Gf group, respectively. However, while McNemar’s test corroborated the differences in the proportions of unique responses given by the DT groups, the Gf groups’ comparisons were not significant in this study. Notably, the highly creative children generated significantly more unique responses, which are probably associated with the flexible organization of the semantic network that might permit an efficient retrieval of remote responses (Kenett and Faust 2019). In this regard, previous studies have provided strong evidence that highly creative individuals employ better search processes that reach further and largely unrelated concepts (Kenett and Austerweil 2016; Gray et al. 2019; Rossmann and Fink 2010). Our results further characterized the network structure of the highly creative children’s group, which showed more flexible associations between concepts. On the contrary, the Gf results were unexpected since, in previous studies, fluid intelligence has also been found to be associated with original performance on DT tasks (Benedek et al. 2012; Silvia and Beaty 2012; Forthmann et al. 2019; Jung and Haier 2013; Karwowski et al. 2016; Carson et al. 2003) even among children (Krumm et al. 2018). 

### 4.3. Implication of the Study, Limitations, and Future Directions

Our work provides an insight into a better understanding of the bottom-up and top-down cognitive mechanisms that explain the individual differences in children’s creativity. Our findings confirm the crucial role of the semantic memory organization in creative performance, and provide a demonstration that this phenomenon can be traced back to childhood. These results suggest that the way in which children memorize could be developed earlier in life.

Whereas the present findings are promising, the limitations need to be taken into consideration. Firstly, despite the sample size of the present study (N = 58, 29 for each group), which has been reasoned to be sufficient for the estimation of the networks as closely related to previous works (Kenett et al. 2016a, 2013), we cannot exclude that here the individuals (overlapping in both analyses) possibly drove the similarity in semantic network observed in the two analyses. Moreover, it is also known that children’s semantic networks are characterized by a smaller size in comparison to the adult sample, suggesting a network with very few nodes and edges (Zortea et al. 2014; Wulff et al. 2019). To overcome these problems, future studies should consider the adoption of a larger sample size in order to avoid individuals’ overlap between groups and ensure an adequate number of nodes and connections. Moreover, in the present study, we used the Standard Progressive Matrices as an index of top-down processes, although other executive functions—such as shifting and inhibition—have been related to creative thinking capacities both in adults and in children (Avitia and Kaufman 2014; Benedek et al. 2014; Silvia 2015; Frith et al. 2019; Krumm et al. 2018; Arán Filippetti and Krumm 2020). Hence, future scientific investigations should also explore the ways in which the influence of other executive functions associate with creative performance in children’s semantic memory topology.

## 5. Conclusions

The present study applied a network science methodology in order to examine the ways in which DT (bottom-up process) and Gf (top-down process) are related to the structure of semantic networks in children. Albeit preliminary, our results suggest that the structure of semantic memory is related to both creative divergent thinking and fluid intelligence capacities, probing its impact on children’s creative cognition. Notably, we found that, even in the development population, DT corresponds to a more flexible structure of the semantic network, while G*f* corresponds to a more structured semantic network. Thus, the semantic memory system of highly creative children and highly intelligent children appears to account for the efficient information processing balancing between a rigid and chaotic network organization, which in turn may lead to original and appropriate solutions to a given problem (Faust and Kenett 2014). Altogether, these results support and extend previous findings (Benedek et al. 2017; Kenett et al. 2014, 2016; Rossmann and Fink 2010), and have major implications for the encouragement of the development of creativity in the field of education. Finally, we also corroborated the network science methodology as a valid approach to study creative cognition in the developmental population.

## Figures and Tables

**Table 1:** Correlations between the three divergent thinking indices and overall divergent thinking.

AUT Variables	Flexibility	Fluency	CreativitySum	ODT
Flexibility	-	0.76 ***	0.80 ***	0.94 ***
Fluency	0.76 ***	-	0.95 ***	0.70 ***
CreativitySum	0.80 ***	0.95 ***	-	0.83 ***
ODT	0.94 ***	0.70 ***	0.83 ***	-

Note. (***) *p* = < 0.001.

**Table 2:** Descriptive and inferential statistics of the groups’ dependent variables.

	DT			G*f*		
	Low (N = 29)	High (N = 29)		Low (N = 29)	High (N = 29)	
Variables	M (SD)	M (SD)	t (p)	M (SD)	M (SD)	t (p)
WPM	11.03 (2.03)	10.65 (1.61)	−0.789	10.48 (1.94)	11.21 (1.66)	1.53
VOC	13.1 (1.45)	13.24 (1.12)	0.405	13.03 (1.45)	13.31 (1.10)	0.814
SVF	9.86 (1.41)	10.59 (2.23)	1.480	9.86 (1.57)	10.59 (2.11)	1.480
G*f* (SPM)	39.07 (6.14)	40.07 (6.47)	0.604	34.55 (4.72)	44.59 (3.11)	10.224 ***
DT (ODT)	3.99 (0.92)	6.49 (0.74)	11.464 ***	5.21 (1.50)	5.28 (1.54)	0.169
Gender (F/M)	15/14	11/18		16/13	10/19	

Note. WPM = Words per minute; VOC = Vocabulary knowledge; SVF = Semantic Verbal Fluency; SPM = Standard Progressive Matrices; ODT = Overall Divergent Thinking. Statistical significance *p* ‘***’ < 0.001; ‘ns’ not significant.

**Table 3:** Empirical and random measures of the DT semantic networks.

Measures	Low	High	Random Network
Average Shortest Path Length (ASPL)	2.36	2.13	2.10 (0.39)
Clustering Coefficient (CC)	0.73	0.74	0.18 (.04)
Modularity (Q)	0.45	0.43	0.29 (.02)
Small-world-ness (S)	2.79	2.95	0.99 (0.15)

Note. Random Network = simulated sampling distribution mean and standard deviation (in parenthesis). All *p* = < 0.001.

**Table 4:** Partial network bootstrapped results for the DT groups.

		Node Remaining				
Network Measures		90% (df = 1998)	80% (df = 1998)	70% (df = 1998)	60% (df = 1998)	50% (df = 1998)
ASPL	*t*	52.662 ***	40.983 ***	26.26 ***	15.742 ***	9.122 ***
	*d*	2.35	1.83	1.17	0.70	0.41
CC	*t*	−37.332 ***	−27.953 ***	−14.812 ***	−5.151 ***	0.926 (ns)
	*d*	1.67	1.25	0.66	0.23	0.04
Q	*t*	22.808 ***	20.432 ***	16.705 ***	12.589 ***	9.141 ***
	*d*	1.02	0.91	0.75	0.56	0.42
S	*t*	−47.035 ***	−31.507 ***	−19.677 ***	−11.548 ***	−8.063 ***
	*d*	2.10	1.41	0.88	0.52	0.36

Note. 1000 samples were generated for each percentage of nodes remaining. The t-statistics and Cohen’s d values are presented (Cohen 1992). Cohen’s d effect sizes: 0.20, small; 0.50, moderate; 0.80, large; 1.10, very large. Negative t-statistics denote the high DT group having lower values than the low DT group. Statistical significance: *p* ‘***’ < 0.001; ‘ns’ not significant. CC = Clustering Coefficient; ASPL = Average Shortest Path Length; Q = modularity index; S = small-world-ness.

**Table 5:** Empirical and random measures of the Gf semantic networks.

Measures	Low	High	Random Network
Average Shortest Path Length (ASPL)	2.10	2.21	2.14 (0.04)
Modularity (Q)	0.40	0.41	0.30 (0.01)

Note. Random Network = simulated sampling distribution mean and standard deviation (in parenthesis). All *p* = < 0.001.

**Table 6:** Partial network bootstrapped results for the Gf groups.

		Node Remaining				
Network Measures		90% (df = 1998)	80% (df = 1998)	70% (df = 1998)	60% (df = 1998)	50% (df = 1998)
ASPL	*t*	−8.662 ***	−4.442 ***	−5.036 ***	−6.687 ***	−1.355 (ns)
	*d*	0.39	0.20	0.22	0.30	0.06
Q	*t*	−16.554 ***	−10.964 ***	−10.738 ***	−9.869 ***	−6.171 ***
	*d*	0.74	0.49	0.48	0.44	0.28

Note. 1000 samples were generated for each percentage of nodes remaining. The t-statistics and Cohen’s d values are presented (Cohen 1992). Cohen’s d effect sizes: 0.20, small; 0.50, moderate; 0.80, large; 1.10, very large. Negative t-statistics denote the high Gf group having lower values than the low G*f* group. Statistical significance *p* ‘***’ < 0.001; ‘ns’ not significant. ASPL = Average Shortest Path Length; Q = modularity index.

## References

[B1-jintelligence-08-00043] Abraham Anna, Bubic Andreja (2015). Semantic Memory as the Root of Imagination. Frontiers in Psychology.

[B2-jintelligence-08-00043] Acar Selcuk, Runco Mark A. (2014). Assessing Associative Distance Among Ideas Elicited by Tests of Divergent Thinking. Creativity Research Journal.

[B3-jintelligence-08-00043] Agresti Alan (2003). Categorical Data Analysis.

[B4-jintelligence-08-00043] Amabile Teresa M. (1983). The Social Psychology of Creativity: A Componential Conceptualization. Journal of Personality and Social Psychology.

[B5-jintelligence-08-00043] Amundson Susan J. (1995). Evaluation Tool of Children’s Handwriting (ETCH).

[B6-jintelligence-08-00043] Anderson John R. (2013). A Spreading Activation Theory of Memory. Readings in Cognitive Science: A Perspective from Psychology and Artificial Intelligence.

[B7-jintelligence-08-00043] Arán Filippetti Vanessa, Krumm Gabriela (2020). A Hierarchical Model of Cognitive Flexibility in Children: Extending the Relationship between Flexibility, Creativity and Academic Achievement. Child Neuropsychology.

[B8-jintelligence-08-00043] Ardila Alfredo, Ostrosky-Solís Feggy, Bernal Byron (2006). Cognitive Testing toward the Future: The Example of Semantic Verbal Fluency (ANIMALS). International Journal of Psychology.

[B9-jintelligence-08-00043] Avitia Maria J., Kaufman James C. (2014). Beyond g and c: The Relationship of Rated Creativity to Long-Term Storage and Retrieval (Glr). Psychology of Aesthetics, Creativity, and the Arts.

[B10-jintelligence-08-00043] Baronchelli Andrea, Ferrer-i-Cancho Ramon, Pastor-Satorras Romualdo, Chater Nick, Christiansen Morten H. (2013). Networks in Cognitive Science. Trends in Cognitive Sciences.

[B11-jintelligence-08-00043] Batey Mark, Furnham Adrian, Safiullina Xeniya (2010). Intelligence, General Knowledge and Personality as Predictors of Creativity. Learning and Individual Differences.

[B12-jintelligence-08-00043] Beaty Roger E., Christensen Alexander P., Benedek Mathias, Silvia Paul J., Schacter Daniel L. (2017). Creative Constraints: Brain Activity and Network Dynamics Underlying Semantic Interference during Idea Production. NeuroImage.

[B13-jintelligence-08-00043] Beaty Roger E., Silvia Paul J. (2012). Why Do Ideas Get More Creative across Time? An Executive Interpretation of the Serial Order Effect in Divergent Thinking Tasks. Psychology of Aesthetics, Creativity, and the Arts.

[B14-jintelligence-08-00043] Beaty Roger E., Silvia Paul J., Nusbaum Emily C., Jauk Emanuel, Benedek Mathias (2014). The Roles of Associative and Executive Processes in Creative Cognition. Memory and Cognition.

[B15-jintelligence-08-00043] Beckage Nicole M., Smith Linda B., Hills Thomas (2010). Semantic Network Connectivity Is Related to Vocabulary Growth Rate in Children Trajectories of Early Vocabulary Growth. Proceedings of the Annual Meeting of the Cognitive Science Society.

[B16-jintelligence-08-00043] Beckage Nicole, Smith Linda, Hills Thomas (2011). Small Worlds and Semantic Network Growth in Typical and Late Talkers. PLoS ONE.

[B17-jintelligence-08-00043] Benedek Mathias, Franz Fabiola, Heene Moritz, Neubauer Aljoscha C. (2012). Differential Effects of Cognitive Inhibition and Intelligence on Creativity. Personality and Individual Differences.

[B18-jintelligence-08-00043] Benedek Mathias, Jauk Emanuel, Christoff Kalina, Fox Kieran C. R. (2018). Spontaneous and Controlled Processes in Creative Cognition. The Oxford Handbook of Spontaneous Thought: Mind-Wandering, Creativity, and Dreaming.

[B19-jintelligence-08-00043] Benedek Mathias, Jauk Emanuel, Sommer Markus, Arendasy Martin, Neubauer Aljoscha C. (2014). Intelligence, Creativity, and Cognitive Control: The Common and Differential Involvement of Executive Functions in Intelligence and Creativity. Intelligence.

[B20-jintelligence-08-00043] Benedek Mathias, Kenett Yoed N., Umdasch Konstantin, Anaki David, Faust Miriam, Neubauer Aljoscha C. (2017). How Semantic Memory Structure and Intelligence Contribute to Creative Thought: A Network Science Approach. Thinking and Reasoning.

[B21-jintelligence-08-00043] Bertail Patrice (1997). Second-Order Properties of an Extrapolated Bootstrap without Replacement under Weak Assumptions. Bernoulli.

[B22-jintelligence-08-00043] Bijvoet-van den Berg Simone, Hoicka Elena (2014). Individual Differences and Age-Related Changes in Divergent Thinking in Toddlers and Preschoolers. Developmental Psychology.

[B23-jintelligence-08-00043] Bilder Robert M., Knudsen Kendra S. (2014). Creative Cognition and Systems Biology on the Edge of Chaos. Frontiers in Psychology.

[B24-jintelligence-08-00043] Borean Michela, Paciulli Giulia, Bravar Laura, Zoia Stefania (2012). Test DGM-P-Test per La Valutazione Delle Difficoltà Grafo-Motorie e Posturali Della Scrittura.

[B25-jintelligence-08-00043] Borge-Holthoefer Javier, Arenas Alex (2010). Semantic Networks: Structure and Dynamics. Entropy.

[B26-jintelligence-08-00043] Borodkin Katy, Kenett Yoed N., Faust Miriam, Mashal Nira (2016). When Pumpkin Is Closer to Onion than to Squash: The Structure of the Second Language Lexicon. Cognition.

[B27-jintelligence-08-00043] Cancho R. F. I., Solé R. V. (2001). The Small World of Human Language. Proceedings of the Royal Society B: Biological Sciences.

[B28-jintelligence-08-00043] Carroll John B., Guilford J. P. (1968). The Nature of Human Intelligence. American Educational Research Journal.

[B29-jintelligence-08-00043] Carson Shelley H., Higgins Daniel M., Peterson Jordan B. (2003). Decreased Latent Inhibition Is Associated with Increased Creative Achievement in High-Functioning Individuals. Journal of Personality and Social Psychology.

[B30-jintelligence-08-00043] Charles Robyn E., Runco Mark A. (2001). Developmental Trends in the Evaluative and Divergent Thinking of Children. Creativity Research Journal.

[B31-jintelligence-08-00043] Christensen Alexander, Kenett Yoed (2019). Semantic Network Analysis (SemNA): A Tutorial on Preprocessing, Estimating, and Analyzing Semantic Networks. PsyArXiv.

[B32-jintelligence-08-00043] Christensen Alexander P. (2019). NetworkToolbox: Methods and Measures for Brain, Cognitive, and Psychometric Network Analysis in R. R Journal.

[B33-jintelligence-08-00043] Christensen Alexander P., Kenett Yoed N., Cotter Katherine N., Beaty Roger E., Silvia Paul J. (2018). Remotely Close Associations: Openness to Experience and Semantic Memory Structure. European Journal of Personality.

[B34-jintelligence-08-00043] Cohen Jacob (1992). power primer. Psychological Bulletin.

[B35-jintelligence-08-00043] Collins Allan M, Loftus Elizabeth F (1975). A Spreading-Activation Theory of Semantic Processing. Psychological Review.

[B36-jintelligence-08-00043] Cropley Arthur J. (2000). Defining and Measuring Creativity: Are Creativity Tests Worth Using?. Roeper Review.

[B37-jintelligence-08-00043] Deyne Simon De, Navarro Danielle J, Perfors Amy, Brysbaert Marc, Storms Gert (2019). Measuring the Associative Structure of English: The ‘Small World of Words’ Norms for Word Association. Behavior Resarch Methods.

[B38-jintelligence-08-00043] Diamond Adele (2013). Executive Functions. Annual Review of Psychology. Annual Reviews of psychology.

[B39-jintelligence-08-00043] Diedrich Jennifer, Benedek Mathias, Jauk Emanuel, Neubauer Aljoscha C. (2015). Are Creative Ideas Novel and Useful?. Psychology of Aesthetics, Creativity, and the Arts.

[B40-jintelligence-08-00043] Dietrich Arne (2004). The Cognitive Neuroscience of Creativity. Psychonomic Bulletin and Review.

[B41-jintelligence-08-00043] Donovan Loretta, Green Tim D., Mason Candice (2014). Examining the 21st Century Classroom: Developing an Innovation Configuration Map. Journal of Educational Computing Research.

[B42-jintelligence-08-00043] Dreu Carsten K. W. de, Nijstad Bernard A., Baas Matthijs, Wolsink Inge, Roskes Marieke (2012). Working Memory Benefits Creative Insight, Musical Improvisation, and Original Ideation through Maintained Task-Focused Attention. Personality and Social Psychology Bulletin.

[B43-jintelligence-08-00043] Dubossarsky Haim, Deyne Simon De, Hills Thomas T. (2017). Quantifying the Structure of Free Association Networks across the Life Span. Developmental Psychology.

[B44-jintelligence-08-00043] Efron Bradley (1979). Computers and the Theory of Statistics: Thinking the Unthinkable. SIAM Review.

[B45-jintelligence-08-00043] Epskamp Sacha, Borsboom Denny, Fried Eiko I. (2018). Estimating Psychological Networks and Their Accuracy: A Tutorial Paper. Behavior Research Methods.

[B46-jintelligence-08-00043] Erdos P., Rényi A. (2011). On the Evolution of Random Graphs. The Structure and Dynamics of Networks.

[B47-jintelligence-08-00043] Eysenck H. J., Thurstone L. L. (1973). Primary Mental Abilities. The Measurement of Intelligence.

[B48-jintelligence-08-00043] Faust Miriam, Kenett Yoed N. (2014). Rigidity, Chaos and Integration: Hemispheric Interaction and Individual Differences in Metaphor Comprehension. Frontiers in Human Neuroscience.

[B49-jintelligence-08-00043] Forthmann Boris, Jendryczko David, Scharfen Jana, Kleinkorres Ruben, Benedek Mathias, Holling Heinz (2019). Creative Ideation, Broad Retrieval Ability, and Processing Speed: A Confirmatory Study of Nested Cognitive Abilities. Intelligence.

[B50-jintelligence-08-00043] Fortunato Santo (2010). Community Detection in Graphs. Physics Reports.

[B51-jintelligence-08-00043] Frith Emily, Elbich Daniel Benjamin, Christensen Alexander, Rosenberg Monica, Chen Qunlin, Kane Michael, Silvia Paul, Seli Paul, Beaty Roger (2019). Intelligence and Creativity Share a Common Cognitive and Neural Basis. Journal of Experimental Psychology: General.

[B52-jintelligence-08-00043] Gajda Aleksandra, Karwowski Maciej, Beghetto Ronald A. (2017). Creativity and Academic Achievement: A Meta-Analysis. Journal of Educational Psychology.

[B53-jintelligence-08-00043] Goñi Joaquín, Arrondo Gonzalo, Sepulcre Jorge, Martincorena Iñigo, De Mendizábal Nieves Vélez, Corominas-Murtra Bernat, Bejarano Bartolomé (2011). The Semantic Organization of the Animal Category: Evidence from Semantic Verbal Fluency and Network Theory. Cognitive Processing.

[B54-jintelligence-08-00043] Gottfredson Linda S. (1997). Why g Matters: The Complexity of Everyday Life. Intelligence.

[B55-jintelligence-08-00043] Gralewski Jacek, Gajda Aleksandra, Wiśniewska Ewa, Lebuda Izabela, Jankowska Dorota M. (2017). Slumps and Jumps: Another Look at Developmental Changes in Creative Abilities. Creativity. Theories–Research-Applications.

[B56-jintelligence-08-00043] Gray Kurt, Anderson Stephen, Chen Eric Evan, Kelly John Michael, Christian Michael S., Patrick John, Huang Laura, Kenett Yoed N., Lewis Kevin (2019). ‘Forward Flow’: A New Measure to Quantify Free Thought and Predict Creativity. American Psychologist.

[B57-jintelligence-08-00043] Guilford J. P. (1950). Creativity. American Psychologist.

[B58-jintelligence-08-00043] Guilford J. P. (1967). Creativity: Yesterday, Today and Tomorrow. The Journal of Creative Behavior.

[B59-jintelligence-08-00043] Guilford Joy Paul (1959). Three Faces of Intellect. American Psychologist.

[B60-jintelligence-08-00043] He Li, Kenett Yoed N., Zhuang Kaixiang, Liu Cheng, Zeng Rongcan, Yan Tingrui, Huo Tengbin, Qiu Jiang (2020). The Relation between Semantic Memory Structure, Associative Abilities, and Verbal and Figural Creativity. Thinking and Reasoning.

[B61-jintelligence-08-00043] Humphries Mark D., Gurney Kevin (2008). Network ‘Small-World-Ness’: A Quantitative Method for Determining Canonical Network Equivalence. PLoS ONE.

[B62-jintelligence-08-00043] Jauk Emanuel, Benedek Mathias, Dunst Beate, Neubauer Aljoscha C. (2013). The Relationship between Intelligence and Creativity: New Support for the Threshold Hypothesis by Means of Empirical Breakpoint Detection. Intelligence.

[B63-jintelligence-08-00043] John, Raven Jean (2003). Raven Progressive Matrices. Handbook of Nonverbal Assessment.

[B64-jintelligence-08-00043] Jung Rex E., Haier Richard J., Vartanian O., Bristol A. S., Kaufman J. (2013). Creativity and Intelligence: Brain Networks That Link and Differentiate the Expression of Genius. Neuroscience of Creativity.

[B65-jintelligence-08-00043] Jung Rex E., Wertz Christopher J., Meadows Christine A., Ryman Sephira G., Vakhtin Andrei A., Flores Ranee A. (2015). Quantity Yields Quality When It Comes to Creativity: A Brain and Behavioral Test of the Equal-Odds Rule. Frontiers in Psychology.

[B66-jintelligence-08-00043] Karuza Elisabeth A., Thompson-Schill Sharon L., Bassett Danielle S. (2016). Local Patterns to Global Architectures: Influences of Network Topology on Human Learning. Trends in Cognitive Sciences.

[B67-jintelligence-08-00043] Karwowski Maciej, Dul Jan, Gralewski Jacek, Jauk Emanuel, Jankowska Dorota M., Gajda Aleksandra, Chruszczewski Michael H., Benedek Mathias (2016). Is Creativity without Intelligence Possible? A Necessary Condition Analysis. Intelligence.

[B68-jintelligence-08-00043] Kenett Yoed N. (2018). Going the Extra Creative Mile: The Role of Semantic Distance in Creativity - Theory, Research, and Measurement. The Cambridge Handbook of the Neuroscience of Creativity.

[B69-jintelligence-08-00043] Kenett Yoed N., Anaki David, Faust Miriam (2014). Investigating the Structure of Semantic Networks in Low and High Creative Persons. Frontiers in Human Neuroscience.

[B70-jintelligence-08-00043] Kenett Yoed N., Austerweil Joseph L (2016). Examining Search Processes in Low and High Creative Individuals with Random Walks. Proceedings of the Annual Meeting of the Cognitive Science Society.

[B71-jintelligence-08-00043] Kenett Yoed N., Beaty Roger E., Silvia Paul J., Anaki David, Faust Miriam (2016a). Structure and Flexibility: Investigating the Relation between the Structure of the Mental Lexicon, Fluid Intelligence, and Creative Achievement. Psychology of Aesthetics, Creativity, and the Arts.

[B72-jintelligence-08-00043] Kenett Yoed N., Faust Miriam (2019). A Semantic Network Cartography of the Creative Mind. Trends in Cognitive Sciences.

[B73-jintelligence-08-00043] Kenett Yoed N., Gold Rinat, Faust Miriam (2016b). The Hyper-Modular Associative Mind: A Computational Analysis of Associative Responses of Persons with Asperger Syndrome. Language and Speech.

[B74-jintelligence-08-00043] Kenett Yoed N., Kenett Dror Y., Ben-Jacob Eshel, Faust Miriam (2011). Global and Local Features of Semantic Networks: Evidence from the Hebrew Mental Lexicon. PLoS ONE.

[B75-jintelligence-08-00043] Kenett Yoed N., Levy Orr, Kenett Dror Y., Stanley H. Eugene, Faust Miriam, Havlin Shlomo (2018). Flexibility of Thought in High Creative Individuals Represented by Percolation Analysis. Proceedings of the National Academy of Sciences of the United States of America.

[B76-jintelligence-08-00043] Kenett Yoed N., Wechsler-Kashi Deena, Kenett Dror Y., Schwartz Richard G., Ben-Jacob Eshel, Faust Miriam (2013). Semantic Organization in Children with Cochlear Implants: Computational Analysis of Verbal Fluency. Frontiers in Psychology.

[B77-jintelligence-08-00043] Kim Kyung Hee (2011). The Creativity Crisis: The Decrease in Creative Thinking Scores on the Torrance Tests of Creative Thinking. Creativity Research Journal.

[B78-jintelligence-08-00043] Kleinfeld Judith S. (2002). The Small World Problem. Society.

[B79-jintelligence-08-00043] Krumm Gabriela, Filippetti Vanessa Arán, Gutierrez Marisel (2018). The Contribution of Executive Functions to Creativity in Children: What Is the Role of Crystallized and Fluid Intelligence?. Thinking Skills and Creativity.

[B80-jintelligence-08-00043] Krumm Gabriela, Filippetti Vanessa Arán, Bustos Daniela (2014). Intelligence and Creativity: Correlates among the Constructs through Two Empirical Studies. Universitas Psychologica.

[B81-jintelligence-08-00043] Lee Christine S., Therriault David J. (2013). The Cognitive Underpinnings of Creative Thought: A Latent Variable Analysis Exploring the Roles of Intelligence and Working Memory in Three Creative Thinking Processes. Intelligence.

[B82-jintelligence-08-00043] Lewis Theodore (2009). Creativity in Technology Education: Providing Children with Glimpses of Their Inventive Potential. International Journal of Technology and Design Education.

[B83-jintelligence-08-00043] Marupaka Nagendra, Iyer Laxmi R., Minai Ali A. (2012). Connectivity and Thought: The Influence of Semantic Network Structure in a Neurodynamical Model of Thinking. Neural Networks.

[B84-jintelligence-08-00043] Marupaka Nagendra, Minai Ali A. (2011). Connectivity and Creativity in Semantic Neural Networks. Proceedings of the International Joint Conference on Neural Networks.

[B85-jintelligence-08-00043] Massara Guido Previde, Matteo T. Di, Aste Tomaso (2016). Network Filtering for Big Data: Triangulated Maximally Filtered Graph. Journal of Complex Networks.

[B86-jintelligence-08-00043] McCrae R. R., Arenberg D., Costa P. T. (1987). Declines in Divergent Thinking with Age: Cross-Sectional, Longitudinal, and Cross-Sequential Analyses. Psychology and Aging.

[B87-jintelligence-08-00043] McRae Ken, Jones Michael (2013). 14 Semantic Memory. The Oxford Handbook of Cognitive Psychology.

[B88-jintelligence-08-00043] Mednick Sarnoff (1962). The Associative Basis of the Creative Process. Psychological Review.

[B89-jintelligence-08-00043] Newman M. E. J. (2006). Modularity and Community Structure in Networks. Proceedings of the National Academy of Sciences of the United States of America.

[B90-jintelligence-08-00043] Nusbaum Emily C., Silvia Paul J. (2011). Are Intelligence and Creativity Really so Different? Fluid Intelligence, Executive Processes, and Strategy Use in Divergent Thinking. Intelligence.

[B91-jintelligence-08-00043] Osborn A. F. (1963). Applied Imagination: The Principles and Procedures of Creative Thinking. 1953.

[B92-jintelligence-08-00043] Pan Xuan, Yu Huihong (2018). Different Effects of Cognitive Shifting and Intelligence on Creativity. Journal of Creative Behavior.

[B93-jintelligence-08-00043] Patterson Karalyn, Nestor Peter J., Rogers Timothy T. (2007). Where Do You Know What You Know? The Representation of Semantic Knowledge in the Human Brain. Nature Reviews Neuroscience.

[B94-jintelligence-08-00043] Plucker Jonathan A., Beghetto Ronald A., Dow Gayle T. (2004). Why Isn’t Creativity More Important to Educational Psychologists? Potentials, Pitfalls, and Future Directions in Creativity Research. Educational Psychologist.

[B95-jintelligence-08-00043] Prabhakaran Ranjani, Green Adam E., Gray Jeremy R. (2014). Thin Slices of Creativity: Using Single-Word Utterances to Assess Creative Cognition. Behavior Research Methods.

[B96-jintelligence-08-00043] Pyryt Michael C. (1998). Human Cognitive Abilities: A Survey of Factor Analytic Studies. Gifted and Talented International.

[B97-jintelligence-08-00043] Ritter Simone M., Ferguson Sam (2017). Happy Creativity: Listening to Happy Music Facilitates Divergent Thinking. PLoS ONE.

[B98-jintelligence-08-00043] Ritter Simone M., Mostert Nel (2017). Enhancement of Creative Thinking Skills Using a Cognitive-Based Creativity Training. Journal of Cognitive Enhancement.

[B99-jintelligence-08-00043] Rossmann Eva, Fink Andreas (2010). Do Creative People Use Shorter Associative Pathways?. Personality and Individual Differences.

[B100-jintelligence-08-00043] Runco Mark A., Jaeger Garrett J. (2012). The Standard Definition of Creativity. Creativity Research Journal.

[B101-jintelligence-08-00043] Runco Mark A. (1994). Problem Finding, Problem Solving, and Creativity.

[B102-jintelligence-08-00043] Said-Metwaly Sameh, Fernández-Castilla Belén, Kyndt Eva, Noortgate Wim Van den, Barbot Baptiste (2020). Does the Fourth-Grade Slump in Creativity Actually Exist? A Meta-Analysis of the Development of Divergent Thinking in School-Age Children and Adolescents. Educational Psychology Review.

[B103-jintelligence-08-00043] Sak Ugur, Maker C. June (2006). Developmental Variation in Children’s Creative Mathematical Thinking as a Function of Schooling, Age, and Knowledge. Creativity Research Journal.

[B104-jintelligence-08-00043] Schilling Melissa A. (2005). A ‘Small-World’ Network Model of Cognitive Insight. Creativity Research Journal.

[B105-jintelligence-08-00043] Scott Ginamarie, Leritz Lyle E., Mumford Michael D. (2004). The Effectiveness of Creativity Training: A Quantitative Review. Creativity Research Journal.

[B106-jintelligence-08-00043] Shalizi Cosma (2010). Computing Science: The Bootstrap. American Scientist.

[B107-jintelligence-08-00043] Shannon Paul, Markiel Andrew, Ozier Owen, Baliga Nitin S., Wang Jonathan T., Ramage Daniel, Amin Nada, Schwikowski Beno, Ideker Trey (2003). Cytoscape: A Software Environment for Integrated Models of Biomolecular Interaction Networks. Genome Research.

[B108-jintelligence-08-00043] Shao Jun (2003). Impact of the Bootstrap on Sample Surveys. Statistical Science.

[B109-jintelligence-08-00043] Siew Cynthia S. Q. (2018). The Orthographic Similarity Structure of English Words: Insights from Network Science. Applied Network Science.

[B110-jintelligence-08-00043] Siew Cynthia S. Q., Wulff Dirk U., Beckage Nicole M., Kenett Yoed N. (2019). Cognitive Network Science: A Review of Research on Cognition through the Lens of Network Representations, Processes, and Dynamics. Complexity.

[B111-jintelligence-08-00043] Silvia Paul J. (2015). Intelligence and Creativity Are Pretty Similar After All. Educational Psychology Review.

[B112-jintelligence-08-00043] Silvia Paul J., Beaty Roger E. (2012). Making Creative Metaphors: The Importance of Fluid Intelligence for Creative Thought. Intelligence.

[B113-jintelligence-08-00043] Smith Gudmund, Carlsson Ingegerd (1985). Creativity in Middle and Late School Years. International Journal of Behavioral Development.

[B114-jintelligence-08-00043] Spitzer Manfred (1997). A Cognitive Neuroscience View of Schizophrenic Thought Disorder. Schizophrenia Bulletin.

[B115-jintelligence-08-00043] Stein Morris I. (1953). Creativity and Culture. Journal of Psychology: Interdisciplinary and Applied.

[B116-jintelligence-08-00043] Steyvers Mark, Tenenbaum Joshua B. (2005). The Large-Scale Structure of Semantic Networks: Statistical Analyses and a Model of Semantic Growth. Cognitive Science.

[B117-jintelligence-08-00043] Rstudio Team (2020). RStudio: Integrated Development for R.

[B118-jintelligence-08-00043] Torrance E. Paul (1968). A Longitudinal Examination of the Fourth Grade Slump in Creativity. Gifted Child Quarterly.

[B119-jintelligence-08-00043] Torrance E. Paul (1972). Career Patterns and Peak Creative Achievements of Creative High School Students Twelve Years Later. Gifted Child Quarterly.

[B120-jintelligence-08-00043] Torrance E. Paul (1981). Predicting the Creativity of Elementary School Children (1958–80)—And the Teacher Who ‘Made a Difference’. Gifted Child Quarterly.

[B121-jintelligence-08-00043] Torrance E Paul, Presbury Jack (1984). The Criteria of Success Used in 242 Recent Experimental Studies of Creativity. Creative Child & Adult Quarterly.

[B122-jintelligence-08-00043] Unsworth Nash, Spillers Gregory J., Brewer Gene A. (2011). Variation in Verbal Fluency: A Latent Variable Analysis of Clustering, Switching, and Overall Performance. Quarterly Journal of Experimental Psychology.

[B123-jintelligence-08-00043] Vernon Philip A. (1983). Speed of Information Processing and General Intelligence. Intelligence.

[B124-jintelligence-08-00043] Wallas Graham (1926). The Art of Thought.

[B125-jintelligence-08-00043] Watts Duncan J., Strogatz Steven H. (1998). Collective Dynamics of ’small-World9 Networks. Nature.

[B126-jintelligence-08-00043] Wijk Bernadette C.M. van, Stam Cornelis J., Daffertshofer Andreas (2010). Comparing Brain Networks of Different Size and Connectivity Density Using Graph Theory. PLoS ONE.

[B127-jintelligence-08-00043] Wulff Dirk, Hills Thomas, Mata Rui (2018). Structural Differences in the Semantic Networks of Younger and Older Adults. PsyArXiv.

[B128-jintelligence-08-00043] Wulff Dirk U., Deyne S. De, Jones Michael N., Mata Rui, Austerweil Joseph L., Baayen R. Harald, Balota David A. (2019). New Perspectives on the Aging Lexicon. Trends in Cognitive Sciences.

[B129-jintelligence-08-00043] Zemla Jeffrey C., Austerweil Joseph L. (2019). Analyzing Knowledge Retrieval Impairments Associated with Alzheimer’s Disease Using Network Analyses. Complexity.

[B130-jintelligence-08-00043] Zortea Maxciel, Menegola Bruno, Villavicencio Aline, Salles Jerusa Fumagalli de (2014). Graph Analysis of Semantic Word Association among Children, Adults, and the Elderly. Psicologia: Reflexao e Critica.

